# Oral Administration of *Lactiplantibacillus plantarum* CCFM8661 Alleviates Dichlorvos-Induced Toxicity in Mice

**DOI:** 10.3390/foods13193211

**Published:** 2024-10-09

**Authors:** Weiwei Ma, Yiyang Zhao, Hang Sun, Ziwei Zhang, Lili Huang

**Affiliations:** College of Pharmacy, Heilongjiang University of Chinese Medicine, Harbin 150006, China; 18504516911@163.com (W.M.); 15835576364@163.com (Y.Z.); wzz15028185622@163.com (H.S.); 15227369952@163.com (Z.Z.)

**Keywords:** pesticide residue, probiotic, dichlorvos, *Lactiplantibacillus plantarum*

## Abstract

Dichlorvos (DDVP) is an organophosphorus pesticide commonly used in agriculture for pest control, which may enter the organism from the food chain and cause harm. This study aimed to investigate the mitigation effect of *Lactiplantibacillus plantarum* CCFM8661 (a strain of the bacteria) on DDVP toxicity. Sixty male mice were randomly divided into five groups including control (saline), model (DDVP), low-dose, medium-dose, and high-dose groups, and alleviating effect was evaluated by determining body weight, pesticide residues, oxidative stress, and inflammation, and by histological analysis. The results showed that compared with the model group, body weight and acetylcholinesterase activity, and SOD, CAT, T-AOC, and GSH levels significantly increased, and serum DDVP content, MDA level, IL-1β, and TNF-α significantly decreased after administration of the *L. plantarum* CCFM8661. The study demonstrated that *L. plantarum* CCFM8661 exhibited a significant detoxification effect on pesticide toxicity in mice, providing a theoretical basis for the application of probiotics in mitigating pesticide-induced damage.

## 1. Introduction

Dichlorvos (2,2-dichlorovinyl dimethyl phosphate, DDVP) is an organophosphorus pesticide that accounts for 38% of global pesticide consumption. It is used to enhance agricultural production, control internal and external parasites in livestock, and eliminate insects that threaten homes, public health, and stored goods [1,2]. However, the long-term widespread use of DDVP may leave pesticide residues in water, soil, and agricultural products [3,4,5]. In early studies, it has been reported that exposure to DDVP causes neurological, respiratory, hepatic, and reproductive abnormalities, in addition to endocrine disruption, mutagenicity, and carcinogenicity [6]. DDVP is classified by the WHO as a class 1B “highly hazardous” chemical [7].

Acetylcholinesterase (AChE) is identified as a crucial indication of DDVP poisoning in the human body [8]. The mechanism by which DDVP becomes toxic to the body is primarily due to its ability to inhibit AChE activity. In the normal state, acetylcholine transmits nerve impulses in skeletal muscle, the brain, and other organs, and it is hydrolyzed by AChE to choline and acetyl coenzyme A, which prevents overstimulation and overload of the nervous system, while acetylcholine accumulates in the synapse and disrupts nerve function, eventually leading to death by poisoning after AChE activity is inhibited [1,9,10]. In addition, acetylcholine, serving as a neurotransmitter, is also closely associated with immunity and affects immune function, including the release of inflammatory factors, through the effects of acetylcholine receptors mAChRs and nAChRs on immune cells [11]. Oxidative stress is also an important molecular mechanism of DDVP toxicity [12]. After the DDVP is ingested into the organism, it is transported by the blood, which plays a key role in systemic circulation, to arrive at the appropriate target organs. The liver, as the body’s main organ for metabolic functions and detoxification, is one of the main targets of pesticide toxicity. The toxin is carried by the blood to the liver, and oxidative stress, as the main mechanism, is induced after chronic exposure to DDVP. Oxidative stress is an imbalance between oxidation and antioxidants that leads to damage to membrane lipids, proteins, DNA, and tissues in the body [13,14]. The body repairs damage or directly reduces the pro-oxidant state through antioxidant systems, including enzymatic and non-enzymatic systems. Enzymatic systems include superoxide dismutase (SOD) and catalase (CAT), which scavenge free radicals and reactive oxygen species (ROS), while non-enzymatic systems involve endogenous compounds in the body, as well as exogenous compounds imported into the body, such as glutathione (GSH), flavonoids, and vitamin C, which prevent the formation of ROS and exhibit antioxidant effects [15]. Pesticide exposure may also damage immune cells and interfere with the immune system by inducing mitochondrial dysfunction and endoplasmic reticulum stress, leading to cytokine fluctuations and even the development of immune-mediated diseases [16]. Therefore, the need to mitigate the health hazards of DDVP on the organism with safe and effective methods has become an urgent problem.

At present, common ways to degrade DDVP include chemical degradation and microbial degradation. Though chemical degradation is effective, it has high input costs [17]. In contrast, microbial degradation is an efficient, economical, and safe treatment method [18]. Lactic acid bacteria (LAB) have a vital role in the human intestinal flora and perform a variety of physiological functions, including the regulation of intestinal flora, improvement of intestinal barrier function, and regulation of the immune system [19,20]. *Lactiplantibacillus plantarum* is one of the extremely important LAB, some strains of which have been included in the list of microorganisms with “Qualification for Safety” (QPS) by the European Food Safety Authority (EFSA) [21]. Numerous experimental studies have shown that *L. plantarum* can degrade pesticides in vitro. Kumral et al. found that the degradation rate of chlorpyrifos after 3 days was 96% and 90%, respectively, when *L. plantarum* LB-1 and *L. plantarum* LB-2 were inoculated in MS medium containing chlorpyrifos, while the degradation rate of deltamethrin after 3 days was 24% and 53%, respectively, when they were inoculated in MS medium containing deltamethrin [22]. Similarly, Zhou et al. found four organophosphorus pesticides involving chlorpyrifos, dichlorvos, phosphate, and trichlorfon in sauerkraut were degraded by *L. plantarum* by 96.2–99.7% [23].

Previous studies have indicated that *L. plantarum* CCFM8661 confers comparable effects in ameliorating diarrhea, brain aging, and acne, especially in mitigating the toxic effects of exogenous compounds such as lead, aluminum, and benzopyrene [24,25,26,27,28,29]. However, few studies have reported the mitigating effect of *L. plantarum* on the toxicity caused by DDVP exposure in the organism. In this study, the feasibility of using *L. plantarum* CCFM8661 as a degrading microorganism to assess its ability to attenuate DDVP toxicity, oxidative stress, and fluctuations in immune factors in mice was evaluated.

## 2. Materials and Methods

### 2.1. Materials

*L. plantarum* CCFM8661 was obtained from Microcon Bio-Tech Co., Ltd. (Chengdu, China). SPF (specific-pathogen-free) male BALB/c mice, 8 weeks old, weight 20 ± 2 g, were provided by Liaoning Changsheng Biotechnology Co., Ltd. (Benxi, China). The assay kit was obtained from Solarbio Bioscience and Technology Co., Ltd. (Beijing, China). DDVP (active ingredient content 77.5%) was purchased from Nantong Jiangshan Pesticide Chemical Co., Ltd. (Nantong, China)

### 2.2. Preparation of L. plantarum CCFM8661

The *L. plantarum* CCFM8661 was prepared by daily culture according to the method reported by Zhao et al. [30]. The strain was activated using MRS medium and cultured to logarithmic phase. After being activated twice, the strain was inoculated into 500 mL MRS liquid medium at 2% inoculum volume and incubated at 37 °C for 16 h. The bacterial solution was centrifuged at 5000 rpm for 5 min and collected. It was then resuspended in 0.85% sterile saline for oral administration to mice.

### 2.3. Animal Experiments

**Figure 1:**
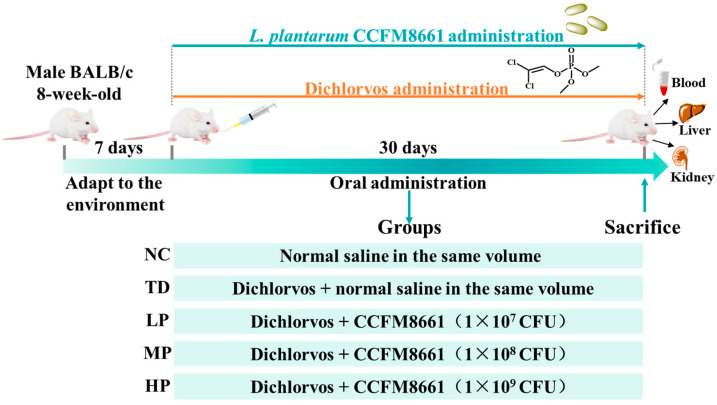
Animal experimental design. NC, TD, LP, MP, and HP indicate normal control, model group, low-dose group, medium-dose group, and high-dose group.

Eight-week-old male BALB/c mice were raised in a controlled environment (*n* = 60). Standard laboratory food and water were provided to the animals and was freely available. Four animals were assigned to each standard cage. The temperature and humidity were maintained at 22 ± 2 °C and 45 ± 5%, respectively, with a 12 h light/dark cycle. After undergoing a 1 week acclimatization period, mice were randomly divided into five groups (n = 12): the normal group (NC), model group (TD), *L. plantarum* CCFM8661 low-dose group (LP), *L. plantarum* CCFM8661 medium-dose group (MP), and *L. plantarum* CCFM8661 high-dose group (HP). Mice in the TD group were orally administered DDVP at a dose of 10 mg/kg BW once daily for 30 days. Mice in the NC group were given equal amounts of saline. Mice in the LP, MP, and HP groups were given DDVP orally at a dose of 10 mg/kg BW, and *L. plantarum* CCFM8661 at a dose of 1 × 10^7^ CFU, 1 × 10^8^ CFU, and 1 × 10^9^ CFU, respectively, once daily for 30 days (Figure 1). The body weight of mice was measured every 5 days. The mice were humanely sacrificed after fasting for 16 h, and blood samples, the liver, and the kidney were rapidly collected for further experimentation. Of note, the dose of 10 mg/kg DDVP was confirmed based on the LOAEL (lowest-observed-adverse-effect level), in accordance with the U.S. Environmental Protection Agency’s level for subchronic exposure [31,32].

The Northeast Agricultural University animal care and welfare committee examined and approved all experiments, which included human care for all the mice, under the approved protocol number NEAUEC20230432. All animal experiments were carried out in accordance with the U.K. Animals (Scientific Procedures) Act, 1986, and associated guidelines, the EU Directive 2010/63/EU for animal experiments, and the National Research Council’s Guide for the Care and Use of Laboratory Animals. In addition, all procedures reported in the study and involving animals were in compliance with the ARRIVE guidelines.

### 2.4. Determination of Dichlorvos Level

The DDVP level in serum was determined according to the method reported by Jiang et al., with slight modifications [33]. The collected blood was centrifuged to acquire serum (3000 r/min, 15 min, 4 °C), and mixed with acetonitrile. High-performance liquid chromatography (HPLC) was used to determine the levels of DDVP.

### 2.5. AChE Activity Assay

AchE catalyzes the hydrolysis of acetylcholine to form choline, which interacts with disulfide-tonitrobenzoic acid (DTNB) to form 5-mercapto-nitrobenzoic acid (TNB); TNB has an absorption peak at 412 nm. AChE activity was measured using an AchE assay kit according to the method reported by Chen et al. [34]. The enzyme activity was calculated by mixing the samples with the reagents according to the manufacturer’s instructions and recording the absorbance at 412 nm using a SpectraMax i3x multi-function microplate reader (MEGU Molecular Instruments (Shanghai) Co., Ltd., Shanghai, China).

### 2.6. Antioxidant Capacity Assay

The blood samples collected were centrifuged at 3000 rpm/min for 15 min at 4 °C to isolate the serum in the form of a clear supernatant for further determination of enzyme activity. The liver was prepared as a 10% homogenate using saline for the next step of the experiment. The SOD activity, CAT activity, MDA (malondialdehyde) level, T-AOC, and glutathione were determined through a procedure provided by a kit obtained from Solarbio Bioscience and Technology Co., Ltd. (Beijing, China). The SOD activity was analyzed based on the principle that SOD inhibits the formation of methyl filth by scavenging O_2_^−^ and the absorbance was recorded at 560 nm. The CAT activity was determined according to the rate of H_2_O_2_ reduction at 240 nm. The MDA level was assayed by recording the absorbance at 532 nm of the trimethyl complex synthesized from MDA and thiobarbituric acid. An assay of reduced glutathione (GSH) was carried out by measuring the absorbance of a complex formed from 5,5′-dithiobis-2-nitrobenzoic acid (DTNB) and GSH at 412 nm.

### 2.7. Cytokine Level Assay

According to a previous study performed by Zhao et al., with slight modifications, the level of interleukin-1β (IL-1β) and tumor necrosis factor-α (TNF-α) in the mouse serum were measured by ELISA based on the protocols of the kit manufacturer (Solarbio Bioscience and Technology Co., Ltd., Beijing, China) [35].

### 2.8. Histopathological Examination

Histological analysis was performed according to the method elaborated by Saka et al. [36]. The liver and the kidney samples were taken in 4% formaldehyde and fixed embedded in paraffin. Paraffin sections were mounted on slides and stained with hematoxylin and eosin. The sections were observed under a BX53 from Olympus Corporation (Nagano, Japan) microscope (×40).

### 2.9. Statistical Analysis

All results are presented as mean ± standard deviation (SD). GraphPad Prism 9.5 was used for all figures. The body weight results were analyzed using repeated measures ANOVA followed a by paired comparison test (IBM SPSS Statistics 25, IBM Corporation, Amonk, NY, USA). Results are shown in the Supplementary Materials. Hierarchical cluster analysis (HCA) used R software (version 3.6.3) on all the features of the mice to determine the similarities and differences within and between the different groups. Other results were analyzed using one-way ANOVA followed by Tukey’s multiple comparison test. (GraphPad Prism 9.5, Software, San Diego, CA, USA).

## 3. Results and Discussion

### 3.1. Body Weight

**Figure 2:**
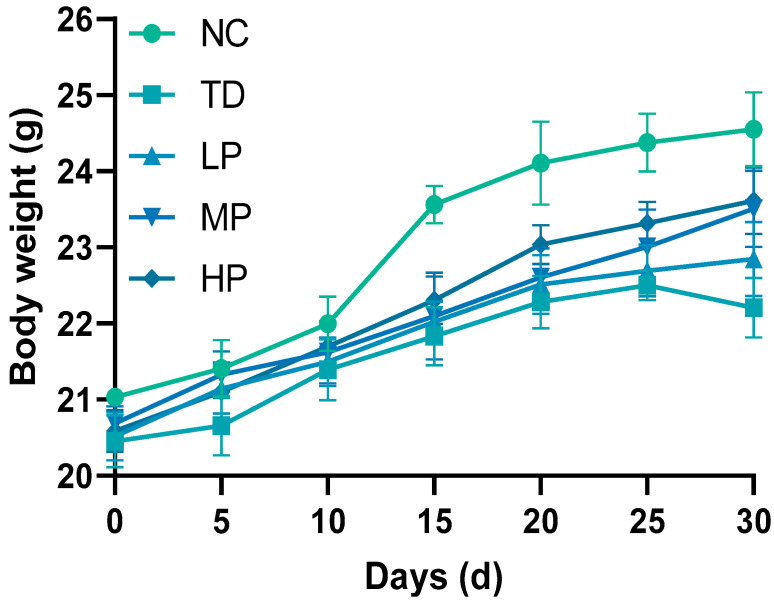
Body weight changes of mice after administration of *L*. *plantarum* CCFM8661 and/or dichlorvos. NC, TD, LP, MP, and HP indicate normal control, model group, low-dose group, medium-dose group, and high-dose group, respectively (*n* = 12).

The growth of mice was reflected by body weight, with changes shown in Figure 2. Compared with the NC group, the body weight of mice in the TD group significantly decreased. After the *L. plantarum* CCFM8661 was supplemented, the body weight of mice in the LP group increased considerably, with the weight gradually increasing with the increase in dosage of the *L. plantarum* CCFM8661. The maximum body weight gain was obtained and this increased by 2.78% in the HP group compared with the TD group. Notably, there was no significant difference in body weight between MP and HP mice (*p* = 0.7860). The decrease in body weight may have been due to the fact that DDVP affects AChE activity, leading to a shortened intestinal transport time, which, in turn, led to a shorter time to digest food, ultimately resulting in an inadequate supply of nutrients to the organism [37]. However, this phenomenon of growth inhibition resulting from DDVP exposure was alleviated by *L. plantarum* CCFM8661. Jiang et al. also obtained a similar result when they found that the reduction in body weight of mice induced by β-cypermethrin was relieved by *Bacillus cereus* GW-01 [33].

### 3.2. Pesticide and AChE Activity

**Figure 3:**
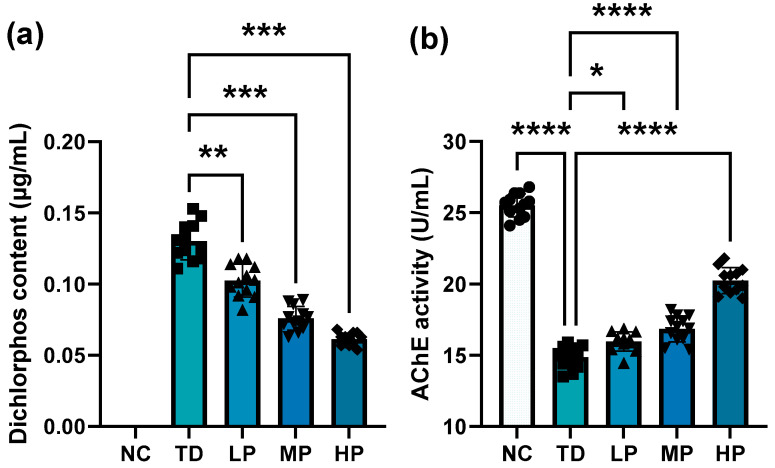
Pesticide (**a**) and AChE activity (**b**) changes in mouse serum after administration of *L*. *plantarum* CCFM8661 and/or dichlorvos. * *p* < 0.05, ** *p* < 0.01, *** *p* < 0.001, and **** *p* < 0.0001 indicated different statistical significances. NC, TD, LP, MP, and HP indicate normal control, model group, low-dose group, medium-dose group, and high-dose group, respectively (*n* = 12).

After ingestion into the body, DDVP diffuses into the bloodstream through the digestive system and then spreads throughout the body with the bloodstream, with part of it remaining in the organs or tissues and most of it excreted in the feces and urine after hepatic detoxification. The concentrations of DDVP in serum were as plotted in Figure 3a; high concentrations of DDVP were detected in mice given DDVP. In the LP group, a significant reduction was observed after *L. plantarum* CCFM8661 intervention, and further decrease was found with an increasing *L. plantarum* CCFM8661 dose. The content of DDVP reduced by 20.8%, 41.5%, and 53.1% in the LP, MP, and HP groups, respectively, compared with the TD group (LP, *p* = 0.0020; MP, *p* = 0.0002; HP, *p* = 0.0002). Similar results were also reported by Jiang et al., who found that the accumulation of β-cypermethrin in lipid, liver, kidney, and feces of mice was alleviated by Bacillus cereus GW-01 [33].

AChE is an enzyme that hydrolyzes the neurotransmitter acetylcholine (ACh) into acetic acid and choline. Ingested DDVP binds to different sites of AChE in the body and inhibits the activity of AChE, which results in the accumulation of ACh at synapses and neuromuscular junctions, leading to uninterrupted stimulation of cholinergic fibers throughout the nervous system, causing overstimulation and death [38]. As indicated in Figure 3b, the AChE activity was obviously inhibited by DDVP, and the inhibition degree was reduced after *L. plantarum* CCFM8661 was orally administrated. Compared with the TD group, the strongest AChE activity was reached in the HP group after oral administration (LP, *p* = 0.0140; MP, *p* < 0.0001; HP, *p* < 0.0001).

### 3.3. Antioxidant Capacity

**Figure 4:**
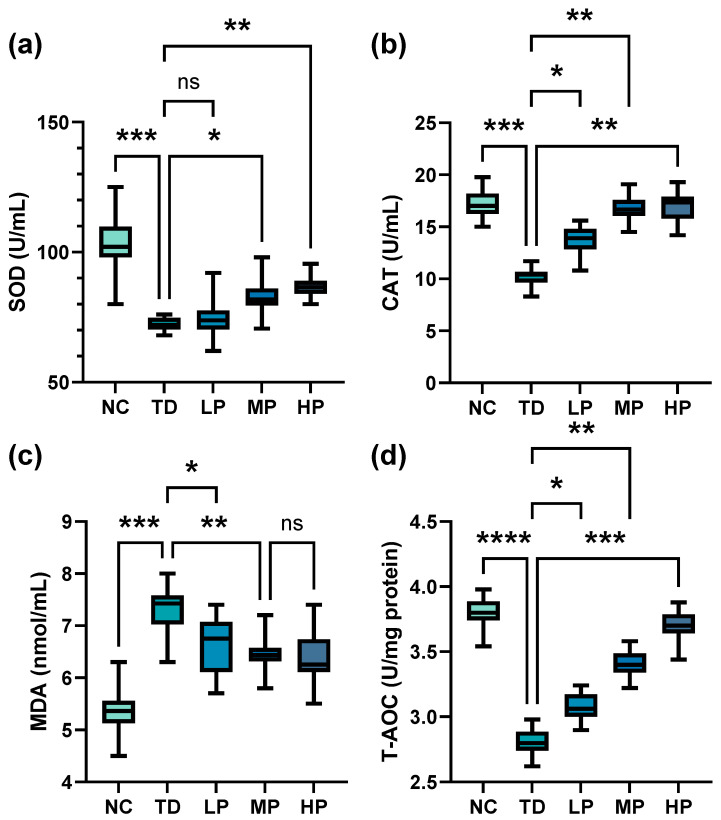
Antioxidant capacity of mouse serum after administration of *L. plantarum* CCFM8661 and/or dichlorvos. (**a**) SOD activity, (**b**) CAT activity, (**c**) MDA, and (**d**) T-AOC, total antioxidant capacity. * *p* < 0.05, ** *p* < 0.01, *** *p* < 0.001, and **** *p* < 0.0001 indicate different statistical significances. Ns, non-significant. The horizontal line in each box represents the median. NC, TD, LP, MP, and HP indicate normal control, model group, low-dose group, medium-dose group, and high-dose group, respectively (*n* = 12).

**Figure 5:**
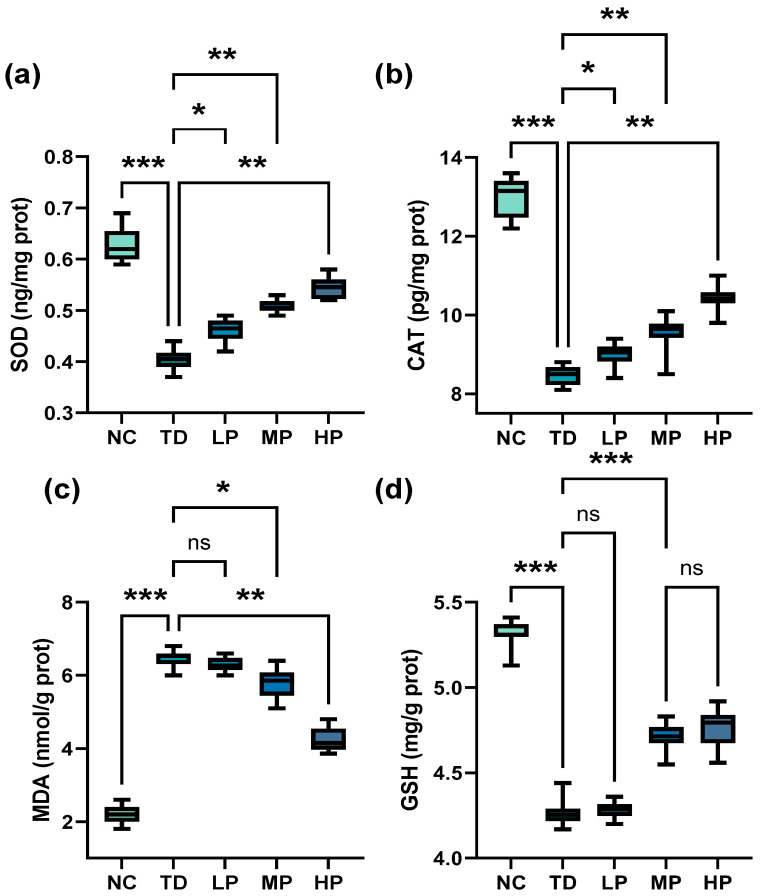
Antioxidant capacity of mouse livers after administration of *L. plantarum* CCFM8661 and/or dichlorvos. (**a**) SOD activity, (**b**) CAT activity, (**c**) MDA, and (**d**) GSH activity. * *p* < 0.05, ** *p* < 0.01, *** *p* < 0.001, indicate different statistical significances. Ns, non-significant. The horizontal line in each box represents the median. NC, TD, LP, MP, and HP indicate normal control, model group, low-dose group, medium-dose group, and high-dose group, respectively (*n* = 12).

SOD is involved in the mutation of the highly reactive superoxide anion (O_2_^−^•) to O_2_ and the less reactive substance H_2_O_2_, and reflects the oxidative stress level of the body [39]. As depicted in Figure 4a and Figure 5a, the SOD activity of the TD group sharply declined in the serum and liver compared with the NC group. When *L. plantarum* CCFM8661 was administrated orally, SOD activity increased significantly with increasing doses of *L. plantarum* CCFM8661. Compared with the TD group, the SOD activity of the mouse serum rose by 2.60%, 14.1%, and 19.8% in the LP, MP, and HP groups, respectively (LP, *p* = 0.9808; MP, *p* = 0.0305; HP, *p* = 0.0018), while the SOD activity of the mouse livers increased by 14.6%, 25.6%, and 34.8%, respectively (LP, *p* = 0.0177; MP, *p* = 0.0047; HP, *p* = 0.0020).

CAT is a crucial antioxidant enzyme that decomposes H_2_O_2_ to O_2_ and H_2_O, resulting in reduced accumulation of radicals [40]. As indicated in Figure 4b and Figure 5b, measurement of CAT activity in all experimental groups showed that CAT activity of mouse serum and liver significantly reduced in the DDVP-exposed group compared with the NC group. With the addition of *L. plantarum* CCFM8661, CAT activity in the TD group increased significantly, and it continued to increase with increasing *L. plantarum* CCFM8661. The strongest CAT activity of mouse serum and liver was obtained in the HP group; it increased by 67.4% and 23.4% relative to TD, respectively (serum, *p* = 0.0032; liver, *p* = 0.0042).

MDA, a lipid peroxidation product produced during oxidative stress, is one of the most frequently measured biomarkers of oxidative stress [41]. As described in Figure 4c and Figure 5c, compared with the control group, there was a significant increase in MDA in the serum and liver of the TD group. With *L. plantarum* CCFM8661 intervening, MDA levels both decreased sharply compared with the TD group, and the minimum MDA content was found in the HP group, in which MDA levels in serum and the liver decreased by 12.6% and 27.1%, respectively (serum, *p* = 0.0034; liver, *p* = 0.0038).

The level of T-AOC reflects the overall oxidative stress level in the body, and excessive free radicals will reduce the level of T-AOC [42]. As shown in Figure 4d, T-AOC in the TD group was significantly lower than that in the NC group. After *L. plantarum* CCFM8661 was consumed, T-AOC dramatically increased and the superior T-AOC was obtained in the HP group (*p* = 0.0002).

GSH plays a key role in detoxifying exogenous compounds, ROS, and free radicals and its main production site is the liver [43,44]. Here, GSH level changes in the liver were determined after DDVP exposure and *L. plantarum* CCFM8661 administration. As depicted in Figure 5d, the GSH was significantly reduced in mice exposed to DDVP (*p* = 0.0002). After oral administration of low-dose *L. plantarum* CCFM8661, no significant difference existed in GSH level compared with the TD group, while a significant increase was observed in the MP group (*p* = 0.0006), and the GSH level continued to increase with increasing *L. plantarum* CCFM8661 dose. Dichlorvos binds to glutathione to produce dimethyl dichlorvos, which is then excreted, thereby reducing glutathione levels. The reduction in GSH levels may also be due to the fact that glutathione binds directly to the electrophilic substances produced by dichlorvos, and, therefore, serves to detoxify exogenous toxic substances [45]. Glutathione is also converted to the oxidized form of oxidized glutathione (GSSG) by ROS and free radicals produced by oxidative stress induced by DDVP exposure [45].

After organophosphorus pesticides enter the organism, a large amount of reactive oxygen species (ROS) is generated, which consumes enzymatic and non-enzymatic antioxidant substances, resulting in oxidative stress. When oxidative stress is greater than the ability of the body to remove it, it causes lipid peroxidation (the oxidized end product is malondialdehyde MDA), and damage to biomolecules such as proteins and DNA [46]. It is obvious that there are various systems in the human and animal body to cope with the oxidative stress generated; one of the important roles is played by the enzyme system, including SOD and CAT [47]. In the present study, DDVP significantly increased the level of MDA and decreased SOD activity, CAT activity, GSH levels, and T-AOC in mice, suggesting that DDVP enhanced the production of free radicals in the serum and the liver of mice, and inhibited antioxidant activity in vivo. Similar findings were found in a recent study reported by Agarwal et al., (2016) who found, after exposure to DDVP, a significant decrease in SOD and CAT, and a significant increase in TBARS (reflecting increased MDA content) in the kidneys of DDVP-exposed mice as compared with the control group, and revealed a possible mechanism of toxicity: DDVP contributes to toxicity by disrupting the activity of certain genes of the enzyme acetylcholinesterase [48]. The antioxidant activity of some probiotics in vivo and in vitro has been confirmed by evidence reported in the literature. In vitro antioxidant activity, the ability to scavenge DPPH and ABTS free radicals of *L. plantarum* KU15120, *L. plantarum* ZJ316, and *L. plantarum* GXL94, has been reported, and the hydroxyl and superoxide scavenging activities of *L. plantarum* ZJ316 and *L. plantarum* GXL94 have also been demonstrated [49,50,51]. In terms of in vivo antioxidants, a previous study reported that after *Lactobacillus acidophilus* intervened, SOD content significantly increased, while MDA in whole serum of Wistar rats raised under thermal stress significantly decreased [52]. In this present study, it was found that SOD and CAT in the serum and the liver of mice significantly increased and MDA levels decreased after *L. plantarum CCFM8661* administration. Similar results were reported by Bouhafs et al., (2015), who found that *L. plantarum* BJ0021 significantly reduced lipid peroxidation in the liver and the kidney tissues and decreased MDA production induced by endosulfan (an organochlorine insecticide) in pregnant rats, proving that *L. plantarum* has effective antioxidant properties and a superior ability to scavenge excess free radicals [53]. This can be attributed to the antioxidant properties of the probiotic strain *L. plantarum* CCFM8661, which helps to overcome exogenous and endogenous oxidative stress. These results indicate that *L. plantarum* CCFM8661 alleviated the decrease in SOD, CAT, GSH, and oxidation to a certain extent.

### 3.4. Cytokine

**Figure 6:**
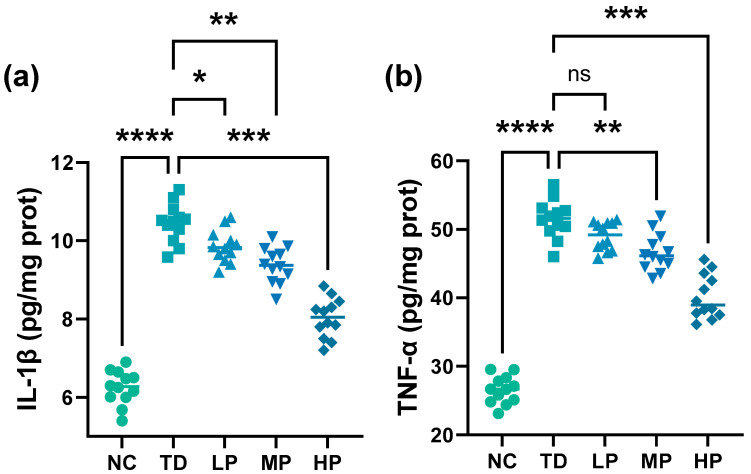
Cytokine level changes of mouse livers after administration of *L*. *plantarum* CCFM8661 and/or dichlorvos. (**a**) IL-1β level and (**b**) TNF-α level. * *p* < 0.05, ** *p* < 0.01, *** *p* < 0.001, and **** *p* < 0.0001 indicate different statistical significances. Ns, non-significant. NC, TD, LP, MP, and HP indicate normal control, model group, low-dose group, medium-dose group, and high-dose group, respectively (*n* = 12).

Organophosphorus pesticides stimulate the body’s immune–inflammatory system after entering the organism, releasing a large number of inflammatory factors. As described in Figure 6, the IL-1β and TNF-α sharply increased in DDVP-administered mice. The IL-1β and TNF-α significantly reduced in *L. plantarum* CCFM8661-administered mice compared with the TD group and minimal levels of IL-1β and TNF-α were acquired in the HP group, decreasing by 23.2% and 22.0%, respectively (IL-1β, *p* = 0.0005; TNF-α, *p* = 0.0001). TNF-α is one of the earliest and most important mediators in the process of inflammatory response; it not only activates neutrophils and lymphocytes but also induces the expression of IL-1β [54]. Immunotoxicity of pesticides to immune cells is associated with their interference with immune cell survival, proliferation, differentiation, and intra-immune cell signaling pathways [55]. Vadhana et al. found that plasma IL-1β, IL-2, IFN-γ, RANTES, and IL-10 levels were elevated in adult rats neonatally exposed to permethrin, compared with control rats [56]. In the present study, *L. plantarum* CCFM8661 intervention significantly reduced the levels of IL-1β and TNF-α and, thereby, reduced the inflammatory response in vivo.

### 3.5. Histological Analysis

**Figure 7:**
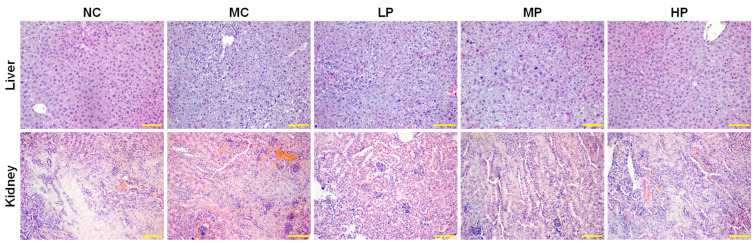
Histological analysis of mouse liver and kidney after administration of *L. plantarum* CCFM8661 and/or dichlorvos. NC, TD, LP, MP, and HP indicate normal control, model group, low-dose group, medium-dose group, and high-dose group, respectively (*n* = 3).

To get a comprehensive understanding of the damage caused by DDVP in these exposed mice and the therapeutic effect of *L. plantarum* CCFM8661 in mice, the livers and the kidneys of the mice were analyzed histologically (Figure 7). As far as the liver tissue was concerned, the hepatic plate structure of mice in the NC group was clear, and the hepatocytes were morphologically intact and arranged in a radial pattern around the central vein. After exposure to DDVP, the liver in the TD group showed a disorganized arrangement of the hepatic plate, hepatic sinusoidal dilatation, swollen hepatocytes, and cytoplasmic vacuolization. In contrast, *L. plantarum* CCFM8661 administration caused hepatoprotective effects, including gradual alignment of the hepatic plate, recovery of the hepatic sinusoids, and reduction in cytoplasmic vacuolization. For the kidney tissues, kidneys in the NC group exhibited normal renal tissues, and no abnormal alterations were observed in the glomerular or tubular regions. However, kidneys in the DDVP-exposed group were caused severe damage, which manifested as atrophy of the renal corpuscle and narrowing of the renal capsule, accompanied by inflammatory cell infiltration. After *L. plantarum* CCFM8661 treatment, renal damage was reduced and the structure was gradually restored to close to normal state. A similar result was reported by Saka et al. where rats exposed to dichlorvos showed destruction of tissue and cellular necrosis of the liver, while the kidney tissue was destroyed with cellular degeneration [36]. Mohapatra et al. also found that fenvalerate-induced hepatocellular necrosis and vascular degeneration were alleviated by mixed supplementation involving *B. subtilis*, *Lactococcus lactis*, and *S. cerevisiae* [57]. The liver plays an important role in the detoxification and excretion of many endogenous and exogenous compounds. The kidney is also one of the important urinary organs whose damage leads to the accumulation of proteins, creatinine, and harmful substances in the blood. The above results suggest that lactic acid bacteria may help animals recover from severe environmental stress by reducing necrosis-related factors [57]. However, the integrity of hepatic and renal tissues was somewhat protected after oral administration of *L. plantarum* CCFM8661 in the present study.

### 3.6. Hierarchical Cluster Analysis

**Figure 8:**
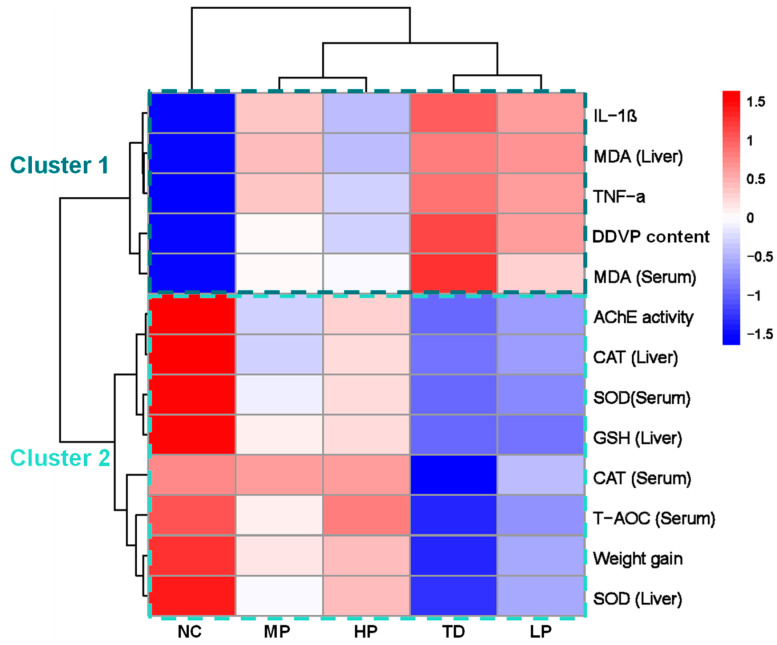
Hierarchical cluster analysis of mice after administration of *L. plantarum* CCFM8661 and/or dichlorvos. NC, TD, LP, MP, and HP indicate normal control, model group, low-dose group, medium-dose group, and high-dose group, respectively.

The results of HCAs are usually displayed in the form of heat maps that stratify the correlation degree within and between different groups [58]. As depicted in Figure 8, in Cluster 1, IL-1β, MDA (liver), TNF-α, DDVP content, and MDA (serum) presented an upregulated trend in the TD, LP, and MP groups, while they exhibited a downregulated trend in the NC and HP groups. In Cluster 2, AChE activity, CAT (liver), SOD (serum), GSH (liver), CAT (serum), T-AOC (serum), weight gain, and SOD (liver) were downregulated in the TD and LP groups, while they were upregulated in the NC and HP groups. Among them, the MP group clustered with the HP group and was similar to the NC group. In addition, the HP group showed a more similar trend to the NC group. As a result, *L. plantarum* CCFM8661 improved the toxicity characteristics of mice in a dose-dependent manner and showed the best remission of toxicity in the HP group.

## 4. Conclusions

The present study demonstrated that DDVP exerts its toxic effects on mice by inhibiting AChE, increasing the level of oxidative stress, decreasing endogenous antioxidant activity, increasing the level of inflammation, and damaging the liver and kidney tissues. However, *L. plantarum* CCFM8661 intervention reduced the accumulation of DDVP in vivo and alleviated DDVP-induced impairment involving body weight loss and suppressed AChE activity, oxidative stress, and inflammatory response in mice, and reduced liver and kidney damage. Therefore, this study provides a new and safe possibility for degrading DDVP, and a new application scenario for *L. plantarum* CCFM8661.

## Data Availability

The data presented in this study are available on request from the corresponding author. The data are not publicly available due to privacy restrictions.

## Supplementary Materials

The following supporting information can be downloaded at: https://www.mdpi.com/article/10.3390/foods13193211/s1, Table S1: The results of repeated measures ANOVA about body weight changes of mice after administration of *L. plantarum* CCFM8661 and/or dichlorvos; Table S2: The results of paired comparison test about body weight changes of mice after administration of *L. plantarum* CCFM8661 and/or dichlorvos.
