# Supplementary material for: Oral Administration of Lactiplantibacillus plantarum CCFM8661 Alleviates Dichlorvos-Induced Toxicity in Mice
Source: Foods. 2024 Oct 9;13(19):3211. doi: 10.3390/foods13193211 (PMC11476327; doi:10.3390/foods13193211)
Supplement: Supplementary file 1 [file foods-13-03211-s001.zip › foods-3157070-supplementary.pdf]

Table S1 The results of repeated measures ANOVA about body weight changes of mice after administration of *L. plantarum* CCFM8661 and/or dichlorvos

	Repeat measurement F test		
	Degree of freedom	F	p
Treated group main effect	4	133.36	p<0.001
Measurement time main effect	4.39	501.97	p<0.001
Treated group * Measurement time	17.56	9.21	p<0.001

Table S2 The results of paired comparison test about body weight changes of mice after administration of *L. plantarum* CCFM8661 and/or dichlorvos

**Paired comparison test between treated groups**		
Treated group	Treated group	p
NC	TD	.000
	LP	.000
	MP	.000
	HP	.000
TD	NC	.000
	LP	.001
	MP	.000
	HP	.000
LP	NC	.000
	TD	.001
	MP	.006
	HP	.000
MP	NC	.000
	TD	.000
	LP	.006
	HP	.786
HP	NC	.000
	TD	.000
	LP	.000
	MP	.786
**Paired comparison test between measurement time**		
Measurement time	Measurement time	p
0 d	5 d	.000
	10 d	.000
	15 d	.000
	20 d	.000
	25 d	.000
	30 d	.000
5 d	0 d	.000
	10 d	.000
	15 d	.000
	20 d	.000
	25 d	.000
	30 d	.000
10 d	0 d	.000
	5 d	.000
	15 d	.000
	20 d	.000
	25 d	.000
	30 d	.000
15 d	0 d	.000
	5 d	.000
	10 d	.000
	20 d	.000
	25 d	.000
	30 d	.000
20 d	0 d	.000
	5 d	.000

	10 d	.000
	15 d	.000
	25 d	.001
	30 d	.000
25 d	0 d	.000
	5 d	.000
	10 d	.000
	15 d	.000
	20 d	.001
	30 d	.760
30 d	0 d	.000
	5 d	.000
	10 d	.000
	15 d	.000
	20 d	.000
	25 d	.760
